# B and T lymphocyte attenuator expression on CD4^+^ T-cells associates with sepsis and subsequent infections in ICU patients

**DOI:** 10.1186/cc13131

**Published:** 2013-11-29

**Authors:** Nicholas J Shubin, Sean F Monaghan, Daithi S Heffernan, Chun-Shiang Chung, Alfred Ayala

**Affiliations:** 1 Department of Surgery at Rhode Island Hospital, Brown University Division of Surgical Research, 593 Eddy Street, Aldrich Building 2nd Floor, Providence, RI 02906, USA

## Abstract

**Introduction:**

Sepsis is a deadly inflammatory condition that often leads to an immune suppressed state; however, the events leading to this state remain poorly understood. B and T lymphocyte attenuator (BTLA) is an immune-regulatory receptor shown to effectively inhibit CD4+ T-cell function. Therefore, our objectives were to determine: 1) if lymphocyte BTLA expression was altered in critically ill patients and experimentally induced septic mice, 2) whether augmented CD4+ T-cell BTLA expression was associated with poor septic patient outcomes, and 3) if BTLA expression affected the CD4+ T-cell apoptotic cell loss observed in the lymphoid organs of septic mice.

**Methods:**

Changes in CD4+ lymphocyte BTLA expression were compared with morbid event development in critically ill ICU patients (11 septic and 28 systemic inflammatory response syndrome subjects). Wild type and BTLA gene deficient mice were utilized to evaluate the expression and role of BTLA in septic lymphocyte apoptotic cell loss.

**Results:**

The observed septic ICU patients had a significantly higher percentage of peripheral blood BTLA+ CD4+ lymphocytes compared with critically ill non-septic individuals. Moreover, the non-septic patients with CD4+ T-cells that were greater than 80% BTLA+ were more susceptible to developing nosocomial infections. Additionally, in general, critically ill patients with CD4+ T-cells that were greater than 80% BTLA+ had longer hospital stays. Comparatively, circulating CD4+ T-cell and B-cell BTLA expression increased in septic mice, which associated with the increased septic loss of these cells. Finally, the loss of these cells and cellular apoptosis induction in primary and secondary lymphoid organs were reversed in BTLA deficient mice.

**Conclusions:**

An increased BTLA+ CD4+ lymphocyte frequency in the observed critically ill non-septic patients was associated with a subsequent infection; therefore, BTLA may act as a biomarker to help determine nosocomial infection development. Additionally, BTLA expression contributed to primary and secondary lymphoid organ apoptotic cell loss in experimentally septic mice; thus, BTLA-induced apoptotic lymphocyte loss may be a mechanism for increased nosocomial infection risk in critically ill patients. This study had a relatively small human subject cohort; therefore, we feel these findings warrant future studies evaluating the use of BTLA as a critically ill patient nosocomial infection biomarker.

## Introduction

Sepsis is a leading killer of critically ill ICU patients [1-3]. Unfortunately, there are currently no effective molecular biological therapeutics approved to treat sepsis [4], and although there appears to be potential in the biomarkers that predict sepsis susceptibility in critically ill patients, overall these are also lacking [4-8]. In the past 15 years it has become accepted that the early events following major trauma and acute sepsis onset cause the adaptive immune system to function at a diminished capacity, which is evident by an inability to clear nosocomial infections and a loss of the delayed-type hypersensitivity response [9,10]. This ‘late septic’ adaptive immune cell suppression is thought to develop in response to an increase in anti-inflammatory mediators, the induction of CD4^+^ T-cell and B-cell loss via apoptosis [11-14], and the actions of immune suppressive cells, such as T-regulatory cells [15-17]. The underlying mechanisms for why these events occur, however, have still yet to be fully defined.

B and T lymphocyte attenuator (BTLA) is a recently characterized co-inhibitory receptor that is known to potently inhibit CD4^+^ T-cell and B-cell function as well as diminish pro-survival signaling in CD4^+^ T cells [18-20]. Co-inhibitory receptors, including programmed death receptor-1 (PD-1), cytotoxic T-lymphocyte antigen-4 (CTLA-4), and BTLA, have also recently gained traction as effective (in the case of anti-CTLA-4; ipilimumab) or potential therapeutic targets in a number of disease states [18,21-23]. These receptors have also been implicated in contributing to sepsis progression, whereby CTLA-4 and PD-1 have been shown to be involved in T-cell apoptosis and dysfunction during experimental sepsis in mice [24-26], while increased PD-1 expression on CD4^+^ T cells correlated with a decreased proliferation capacity in humans [27]. Recently, we have also reported that BTLA [28] and PD-1 [29] contribute to septic morbidity and mortality in mice, while also causing innate inflammatory cell dysfunction during acute sepsis [28,29]. Although BTLA expression on CD4^+^ T cells and B cells has been well documented [18,19], and expression on these cells has been shown to contribute to a number of disease states [30-33], the significance of BTLA expression on lymphocytes during sepsis has yet to be fully addressed. We therefore set out to understand whether BTLA plays a role in driving lymphocyte dysfunction and apoptosis during sepsis.

## Materials and methods

### Patients

Blood was obtained from trauma or surgical ICU patients and was processed for BTLA expression using flow cytometry by investigators who were blinded to the clinical data. All patients classified as having a systemic inflammatory response syndrome (SIRS) response (*n* = 28) or a septic response (*n* = 11), as described previously, were included in the study [3]. While all samples were taken within 24 hours following enrollment in the study, median sampling times were 4 days (range = 0 to 43 days) and 21 days (range = 3 to 83 days) post ICU admittance for the SIRS patients and the septic patients, respectively (Table 1). The development of nosocomial infections, infections acquired at least 72 hours following hospital admittance, were noted following hospital ICU admission, not according to sampling time. All observed sepsis instances were believed to be nosocomial in origin. Additionally, a secondary infection was defined as an additional infection (that occurred following the initial sampling) appearing in a patient who was septic at the time of sampling. Further, a secondary infection was only defined as such if it was caused by a different pathogen. For comparison, blood was also taken from healthy controls (*n* = 6). Full Rhode Island Hospital Institutional Review Board approval was obtained. Consent was obtained from the volunteers for the blood draw and inclusion. Since ICU patient blood was obtained concomitant with the daily laboratory draws, no written consent was necessary; however, the purpose of the study was explained to the participants (assent) prior to inclusion in the study.

**Table 1:** Clinical characteristics of the critically ill ICU patients assessed

	**SIRS (*n*** **= 28)**	**Sepsis (*n*** **= 11)**
Age	59.8 (3.8)	58.4 (7.2)
Male gender	17 (60.7%)	6 (54.5%)
APACHE II score	16.6 (4 to 29)	20.0 (5 to 26)
Patients from trauma ICU	11 (39.3%)	5 (45.4%)
Patients from surgical ICU	17 (60.7%)	6 (54.6%)
Average hospital length of stay (days)	31.1 (5.1)	58.4* (10.5)
Site of infection		
Lung		5 (45.4%)
Blood		2 (18.2%)
Abdominal		1 (9.1%)
Urinary tract		1 (9.1%)
Skin		1 (9.1%)
Catheter		1 (9.1%)
Subsequent nosocomial infection	11 (39.2%)	8 (72.7%)
Site of infection		
Lung	3 (27.3%)	4 (50%)
Abdominal	1 (9.1%)	0 (0%)
Urinary tract	5 (45.4%)	3 (37.5%)
Skin	2 (18.2%)	1 (10%)
Sampling time following ICU admission (days)	4 (0 to 45)	21 (3 to 83)
Days following subsequent infection		
Following ICU admission		31.7 (9 to 94)
Following sampling		22.38 (6 to 49)

Data presented as mean (standard error of the mean), number (%) or median (range). APACHE, Acute Physiology and Chronic Health Evaluation; SIRS, systemic inflammatory response syndrome. *P <0.05 via an unpaired, two-tailed Student’s *t* test.

### Mice

Male, age-matched (8 to 12 weeks of age) wild-type (WT) C57BL/6 and BTLA^-/-^ gene-deficient mice were obtained from Jackson Laboratories (Bar Harbor, ME, USA). All protocols were carried out in compliance with the National Institutes of Health Guide for Animal Use and Care and were approved by the animal welfare committee of Rhode Island Hospital.

### Cecal ligation and puncture

Cecal ligation and puncture (CLP) was used to induce experimental sepsis in mice as described previously [28,34]. The procedures and steps to minimize animal suffering were approved by the Rhode Island Hospital Institutional Animal Care and Use Committee.

### B and T lymphocyte attenuator cellular phenotyping

#### Human subjects

Peripheral blood from critically ill patients and healthy donors was collected in anticoagulant, ethylenediamine tetraacetic acid-containing tubes. Red blood cells were lysed and leukocytes were stained with anti-BTLA (clone J168-540; BD Bioscience, San Jose, CA, USA) and analyzed by flow cytometry, as described previously [28]. CD4^+^ T cells were stained with monoclonal anti-CD4 (clone OKT4; eBioscience, San Diego, CA, USA) following gating on the lymphocyte population and examined using Flowjo analysis software (Tree Star, Ashland, OR, USA).

#### Mice

Peripheral blood leukocytes were isolated 24 hours and 72 hours following CLP or sham surgeries, and red blood cells were lysed as above. Splenocytes were obtained by homogenizing spleens between frosted glass slides followed by red blood cell lysis. BTLA (clone 6 F7; eBioscience) monoclonal antibody was used to assess for surface expression on lymphocyte populations using monoclonal antibodies to CD3 (clone 145-2C11; eBioscience), CD4 (clone RM4-5; Biolegend, San Diego, CA, USA), and B220-hi (RA3-6B2; eBioscience) populations using flow cytometry as described elsewhere [28]. Intracellular FoxP3 staining, following use of the FoxP3 staining kit (eBioscience), was measured with anti-FoxP3 (clone N418; eBioscience). FoxP3 was measured to distinguish that the CD4^+^ T-cell population examined did not contain CD4^+^ T-regulatory cells. The extent of BTLA expression/cell was calculated by determining the mean fluorescent intensity (MFI) divided by the isotype control MFI for standardization. Ten CLP mice and six sham mice were utilized for the phenotyping studies. The MFI experiments were conducted with three to five mice for each group and were then repeated for confirmation of the results.

### Assessment of apoptosis

The spleen and thymus were obtained from WT and BTLA^-/-^ mice 24 hours following the sham and CLP surgical procedures, and were then fixed in 10% formalin, paraffin embedded, sectioned and stained with hematoxylin and eosin or by a terminal deoxynucleotidyl transferase (TUNEL) assay (Roche, Indianapolis, IN, USA) as described previously [28]. The extent of change in the TUNEL staining and the nuclear condensation in the hematoxylin and eosin-stained cells was assessed from images at 20× magnification with a minimum of three fields per organ per sample being quantified using Image J software (National Institutes of Health, Bethesda, MD, USA).

### Statistical analysis

Results are expressed as the mean ± standard error of the mean. Statistical significance of the results presented was determined utilizing the Bonferroni post test following one-way analysis of variants for multiple comparisons, an unpaired two-tailed Student’s *t* test or the Mann–Whitney test for nonparametric data, or the use of a Spearman coefficient following a nonparametric correlation test. The statistical software used was Prism 5.0 (GraphPad Software, Inc., La Jolla, CA, USA). *P* <0.05 was used as a cutoff for significance.

## Results

### Percentage of circulating BTLA^+^CD4^+^ T lymphocytes was higher in the septic compared with the SIRS ICU patients

**Figure 1:**
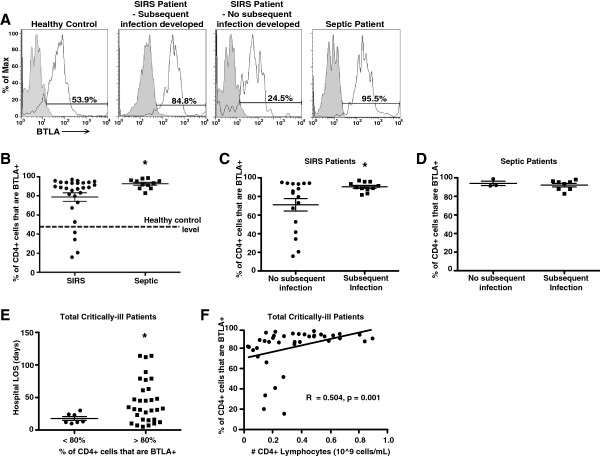
**High BTLA**^**+**^**CD4**^**+ **^**T-lymphocyte percentage associated with poor critically ill ICU patient outcomes.** Systemic inflammatory response syndrome (SIRS) patients that did not develop a subsequent nosocomial infection had a lower BTLA^+^CD4^+^ T-cell percentage compared with SIRS patients that developed a nosocomial infection and patients who were septic at the time of sampling **(A)** (histograms represent CD3^+^CD4^+^ gated lymphocytes). Specifically, a significantly diminished BTLA^+^CD4^+^ lymphocyte percentage was observed in the total SIRS population (*n* = 28) compared with the septic population (*n* = 11) **(B)** (dashed line, mean healthy volunteer BTLA^+^CD4^+^ lymphocyte percentage (*n* = 6); these patients were not sex or aged matched). Importantly, a significantly diminished BTLA^+^CD4^+^ lymphocyte level was also observed in the SIRS patients who did not develop a subsequent infection compared with those that did prior to hospital discharge **(C)**. Additionally, septic patients that went on to develop a secondary nosocomial infection also maintained a high BTLA^+^CD4^+^ lymphocyte percentage **(D)**. Regarding how the BTLA^+^CD4^+^ lymphocyte percentage associated with patient outcomes, the patients with >80% BTLA^+^CD4^+^ lymphocytes had a significantly longer hospital length of stay (LOS) **(E)**. The BTLA^+^CD4^+^ lymphocyte percentage correlated moderately with the circulating CD4^+^ lymphocyte number in the evaluated total critically ill patient pool, suggesting that BTLA expression does not diminish the ability for CD4^+^ lymphocytes to enter circulation **(F)**. Data are mean ± standard error of the mean. *P <0.05 using an unpaired, two-tailed Student’s *t* test. BTLA, B and T lymphocyte attenuator; *R*, Spearman coefficient following a nonparametric correlation test.

The apoptotic loss and diminished functional capacity of CD4^+^ lymphocytes in critically ill septic patients are well known [9,10]. Given BTLA’s role in diminishing CD4^+^ T-cell function [18,31] and pro-survival signaling [20], we looked for differences in BTLA expression on these cells from the peripheral blood of septic (*n* = 11) compared with nonseptic/SIRS (*n* = 28) critically ill ICU patients to assess whether increased BTLA expression may be a marker for poor septic outcomes, potentially due to BTLA-induced CD4^+^ T-cell apoptosis and/or diminished function (patient characteristics are described in Table 1). We found that the percentage of circulating BTLA^+^CD4^+^ lymphocytes was significantly higher in the septic patients compared with the patients with SIRS alone (Figure 1A,B).

An increased percentage of BTLA^+^CD4^+^ T lymphocytes in critically ill patients was associated with an increased incidence of subsequent nosocomial infections and a longer hospital length of stay.

As several recent studies have suggested, co-inhibitor expression contributes to CD4^+^ T-cell loss and dysfunction, while also contributing to septic morbidity [13,32]. We therefore investigated whether or not the observed increase in the percentage of BTLA^+^CD4^+^ lymphocytes in the critically ill patients associated with the development of a subsequent nosocomial infection and an increased hospital length of stay. With regard to the SIRS patients, we found that those patients who developed a subsequent nosocomial infection had a significantly higher percentage of BTLA^+^CD4^+^ lymphocytes than the SIRS patients who did not develop later infections (Figure 1B). Further, these patients developed a subsequent infection at least 6 days following blood sampling (Table 1); therefore, this may have been due to a dysfunction in the adaptive immune response, such as with CD4^+^ T cells. In the septic patients (those who already had an identified source of infection), the correlation between the changes in the percentage of BTLA^+^CD4^+^ lymphocytes and the development of a second subsequent infection during the same hospital stay was lost (Figure 1C). However, one must note that nearly all of the CD4^+^ T cells expressed BTLA in the majority of septic patients who developed a secondary infection, as stated above (Figure 1C). No association was found with the percentage of BTLA^+^CD4^+^ lymphocytes and the patients that died, since only six of the total patients examined died while hospitalized (data not shown). Future studies with a larger patient enrollment will therefore be needed to understand whether an increase in the percentage of BTLA^+^CD4^+^ T cells correlates with death in critically ill SIRS or septic patients. Importantly, we also found that the critically ill patients had a significantly increased hospital length of stay if their CD4^+^ lymphocytes exceeded a threshold level of >80% BTLA^+^ (Figure 1D). This level of BTLA expression was chosen based on the observation that >80% of all septic patients had CD4^+^ T cells that expressed BTLA (Figure 1B).

### Percentage of circulating BTLA^+^CD4^+^ T cells moderately correlated with the number of circulating CD4^+^ T cells

Surprisingly, we observed that an increase in the percentage of CD4^+^ T cells that were BTLA^+^ directly correlated with an increase in CD4^+^ T-cell number (Figure 1E). These data were unexpected because of the inhibitory nature of BTLA on CD4^+^ T cells. Interestingly, a recent article evaluating the nature of BTLA on CD4^+^ T cells in HIV patients found similar results to the data we observed with these critically ill patients [33]. These studies also found that when the percentage of the circulating BTLA^+^CD4^+^ T cells increased, the number of the circulating CD4^+^ T cells also increased. These data suggest that although BTLA is inhibitory in nature, it may also be important for driving CD4^+^ T cells into circulation following SIRS or sepsis induction.

Based on these results, and the results from our previous study whereby we found that BTLA gene-deficient mice were protected from experimental septic morbidity and mortality [34], we evaluated the effects of BTLA on CD4^+^ T cells following experimental sepsis induction in mice.

### BTLA expression increased in intensity on CD4^+^ T cells and B cells following experimental septic challenge in mice

**Figure 2:**
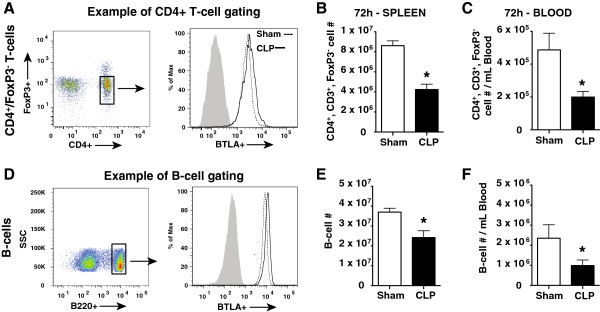
**B and T lymphocyte attenuator expression was induced on CD4**^**+ **^**T cells and B cells 72 hours following cecal ligation and puncture and was associated with the loss of these cells.** Splenic CD4^+^ T-cell **(A)** and B-cell **(D)** B and T lymphocyte attenuator (BTLA) expression was evaluated 72 hours post surgery. As expected, BTLA was induced on CD4^+^ T cells **(A)** and B cells **(D)** (mean fluorescent intensity values presented in Table 2). Concomitantly, a significant decrease in the CD4^+^ T-cell **(B)** and B-cell **(E)** numbers at 72 hours following cecal ligation and puncture (CLP) (*n* = 10) was observed when compared with sham mice (*n* = 6). Additionally, significantly lower peripheral blood CD4^+^ T-cell **(C)** and B-cell **(F)** numbers were also observed at this time point. Data are mean ± standard error of the mean. *P <0.05 using an unpaired, two-tailed Student’s *t* test. Representative flow histograms: filled grey, isotype control; dashed line, sham mouse; black line, CLP mouse.

As with the human subject observational study above, we evaluated changes in the level of BTLA expression on CD4^+^ T cells, as well as B cells, following experimental sepsis induction in mice (CLP) as compared with a sham procedure. Interestingly, at 24 hours and 72 hours following CLP and sham surgeries, we found that nearly all spleen and circulating CD4^+^ T cells and B cells expressed BTLA (Tables 2 and 3 and an example in Figure 2A,D). These findings differed from what was observed in the critically ill patients, however, whereby some of the CD4^+^ T cells from the nonseptic critically ill patients did not express BTLA (Figure 1A). Because nearly all of the mouse CD4^+^ T cells and B cells expressed BTLA following CLP and sham surgeries, we also evaluated the extent of BTLA that was expressed by these cells by assessing the MFI of the BTLA antibody using flow cytometry. We found no changes in the BTLA MFI at 24 hours following the CLP and sham surgeries; however, there was an approximately 1.5-fold increase in the BTLA MFI on both the circulating and splenic CD4^+^ T cells and B cells after 72 hours in the mice subjected to CLP compared with the sham mice (Tables 2 and 3 and representative histograms in Figure 2A,D).

**Table 2:** **CD4**^**+ **^**T cells (CD3**^**+**^**, CD4**^**+**^**, FOXP3**^**–**^**) gained BTLA expression (MFI) 72 hours following CLP surgery**

	**Sham mice (*n*** **= 6)**	**CLP mice (*n*** **= 10)**	***P *** **value**
Blood – 24 hours post surgery			
% BTLA^+^ (SEM)	96.72 (0.65)	93.36 (0.88)	**0.01**
BTLA MFI (SEM)	11.91 (1.07)	10.00 (0.41)	NS
Blood – 72 hours post surgery			
% BTLA^+^ (SEM)	95.80 (1.41)	94.34 (1.70)	NS
BTLA MFI (SEM)	8.79 (0.48)	12.10 (0.66)	**0.02**
Spleen – 24 hours post surgery			
% BTLA^+^ (SEM)	97.67 (0.27)	97.29 (0.50)	NS
BTLA MFI (SEM)	10.89 (0.20)	10.73 (0.76)	NS
Spleen – 72 hours post surgery			
% BTLA^+^ (SEM)	98.44 (0.36)	98.23 (0.90)	NS
BTLA MFI (SEM)	7.43 (0.18)	11.64 (0.64)	**<0.01**

*P* value evaluated via an unpaired, two-tailed Student’s *t* test ± standard error of the mean (SEM); bold values are significant. Mean fluorescent intensity (MFI) experiments were conducted with three to five mice for each group and then repeated for confirmation of the results. BTLA, B and T lymphocyte attenuator; CLP, cecal ligation and puncture; NS, not significant.

**Table 3:** **B cells (B220**^**high**^**) gained BTLA expression (MFI) 72 hours following CLP surgery**

	**Sham mice (*n*** **= 6)**	**CLP mice (*n*** **= 10)**	***P *** **value**
Blood – 24 hours post surgery			
% BTLA^+^ (SEM)	99.98 (0.02)	98.88 (0.25)	**<0.01**
BTLA MFI (SEM)	43.74 (1.74)	37.92 (3.80)	0.24
Blood – 72 hours post surgery			
% BTLA^+^ (SEM)	99.92 (0.02)	98.94 (0.66)	0.31
BTLA MFI (SEM)	48.54 (1.13)	71.15 (2.66)	**<0.01**
Spleen – 24 hours post surgery			
% BTLA^+^ (SEM)	99.83 (0.03)	99.85 (0.02)	0.65
BTLA MFI (SEM)	31.91 (0.60)	33.96 (1.10)	0.16
Spleen – 72 hours post surgery			
% BTLA^+^ (SEM)	99.88 (0.02)	99.86 (0.05)	0.77
BTLA MFI (SEM)	26.33 (2.08)	37.89 (1.76)	**<0.01**

*P* value evaluated via an unpaired, two-tailed Student’s *t* test ± standard error of the mean (SEM); bold values are significant. Mean fluorescent intensity (MFI) experiments were conducted with three to five mice for each group and then repeated for confirmation of the results. BTLA, B and T lymphocyte attenuator; CLP, cecal ligation and puncture.

BTLA gene deficiency ameliorated the onset of cellular apoptosis in primary and secondary lymphoid organs and was associated with the loss of CD4^+^ T cells and B cells following CLP in mice.

**Figure 3:**
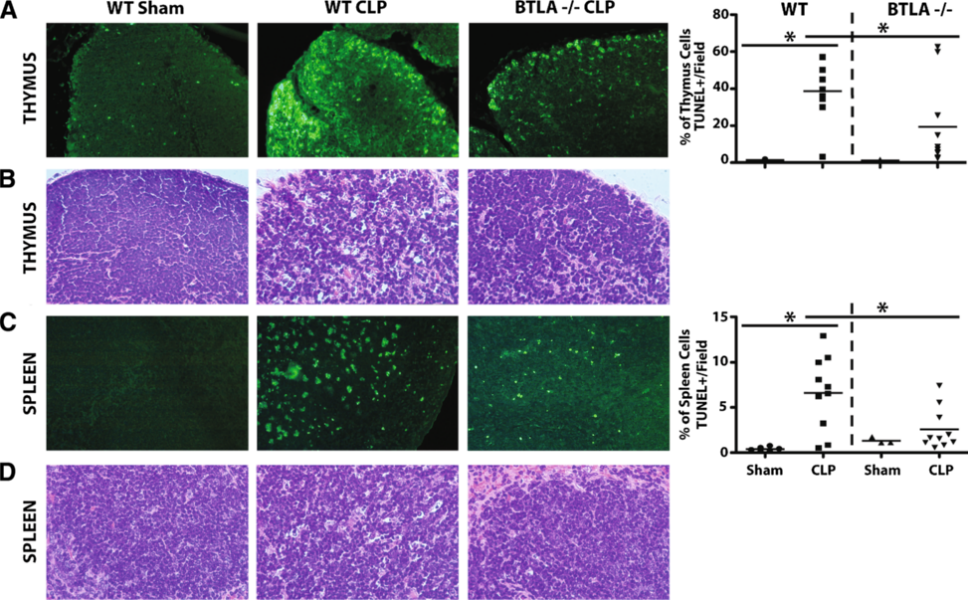
**B and T lymphocyte attenuator expression contributed to increased lymphoid organ cellular apoptosis following cecal ligation and puncture.** Lymphoid cell apoptosis is observed during sepsis and is thought to contribute to infection susceptibility; we therefore evaluated the role of B and T lymphocyte attenuator (BTLA) in this process. Following cecal ligation and puncture (CLP) (24 hours), the wild-type (WT) mice (*n* = 10) were observed to have significantly increased thymic **(A, B)** and splenic **(C, D)** cell apoptosis via increased terminal deoxynucleotidyl transferase (TUNEL) staining **(A, C)** and nuclear condensation (hematoxylin and eosin staining) **(B, D)** when compared with sham mice (*n* = 6). Significantly lower apoptosis levels were observed in the BTLA^-/-^ mice 24 hours post CLP (*n* = 10) compared with the sham mice (*n* = 3) **(A to D)**. Similarly, 24 hours following CLP, the BTLA^-/-^ mice had significantly lower thymic and splenic cell apoptosis levels compared with the observed WT CLP mice **(A to D)**. Data are mean ± standard error of the mean. *P <0.05 using the Bonferroni post test following one-way analysis of variants. At least three fields per organ per sample at 20× magnification were used for the TUNEL staining quantification.

Splenic CD4^+^ T cells and B cells are apoptotically lost in the lymphoid organs of critically ill patients during sepsis [12]. Additionally, other studies have found that BTLA contributes to CD4^+^ T-cell apoptosis in mice [20]; we therefore assessed the contribution of BTLA in lymphocyte apoptosis in our model of sepsis. We observed that BTLA^-/-^ mice were significantly protected from cellular apoptosis (TUNEL staining and assessment for nuclear condensation via hematoxylin and eosin staining) in both the thymus (Figure 3A,B) and the spleen (Figure 3C,D) 24 hours following CLP. Because cell loss is a product of apoptosis, we found that spleen and peripheral blood CD4^+^ T-cell (non-T-regulatory) numbers (Figure 2B,C) and B-cell numbers (Figure 2E,F), 72 hours following CLP, were decreased when compared with the sham mice (gating strategy found in Figure 2A,D). These results strongly suggest that a BTLA-induced apoptotic mechanism contributed, directly or indirectly, to the observed loss of the lymphocytes in the spleen and peripheral blood.

### BTLA^-/-^ mice induced an increased level of CD4^+^ T cells following experimental sepsis induction

**Figure 4:**
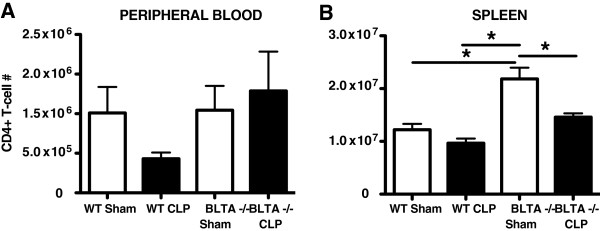
**Following cecal ligation and puncture, a higher circulating CD4**^**+ **^**T-cell level was observed in BTLA**^**-/- **^**mice.** BTLA^-/-^ mice were evaluated to assess whether a lack of B and T lymphocyte attenuator (BTLA) expression on CD4^+^ T cells diminished their capacity to circulate, 24 hours following cecal ligation and puncture (CLP). The level of circulating CD4^+^ T cells, however, was increased following CLP in the BTLA^-/-^ mice **(A)**. When the spleens of the BTLA^-/-^ mice were observed, the level of CD4^+^ T cells in the sham BTLA^-/-^ mice were significantly higher than both the wild-type (WT) sham and CLP mice, as well as the BTLA^-/-^ mice that were subjected to CLP, suggesting that a lack of splenic apoptosis led to a higher level of circulating CD4^+^ T cells **(B)**. Data are mean ± standard error of the mean. *P <0.05 using an unpaired, two-tailed Student’s *t* test.

In Figure 1E we observed that an increase in the percentage of BTLA^+^CD4^+^ lymphocytes associated with an increase in the circulating levels of CD4^+^ lymphocytes. This observation led us to hypothesize that BTLA expression on CD4^+^ T cells may correlate directly with the circulating level of these cells. We therefore evaluated the levels of both circulating and splenic CD4^+^ T cells in WT mice compared with BTLA^-/-^ mice following sham and CLP surgeries. Because nearly all of the circulating mouse CD4^+^ T cells appeared to express BTLA, we could not evaluate whether the addition of BTLA expression following CLP in the WT mice contributed to changes in CD4^+^ T-cell migration out of the secondary lymphoid organs, as it appeared to in the critically ill humans. We thus evaluated BTLA^-/-^ mice to determine whether a lack of BTLA expression led to a decrease in the circulating CD4^+^ T cells following CLP compared with WT mice. We found that, as in septic humans, CLP led to a lower level of circulating CD4^+^ T cells in the WT mice (Figure 4A). We observed no difference, however, in the level of the circulating CD4^+^ T cells in the BTLA^-/-^ mice, 24 hours following CLP compared with sham surgery (Figure 4A). When we evaluated splenic levels of these cells, we observed that BTLA^-/-^ mice had significantly more CD4^+^ T cells following sham surgery compared with the WT mice (Figure 4B). Furthermore, these levels were decreased in the spleens of the BTLA^-/-^ mice following CLP (Figure 4B). Therefore, given that the BTLA^-/-^ sham mice had significantly more splenic CD4^+^ T cells – perhaps due to a lack of BTLA-induced apoptosis (Figure 2; as has been demonstrated in [20], which showed that BTLA^-/-^ T cells have improved survival, but not improved proliferative capacity) – additional studies are needed to evaluate the role of BTLA in CD4^+^ T-cell migration.

## Discussion

Sepsis is a deadly condition and its onset has been thought to be the result of a hyper-inflammatory process that often leads to an immune-suppressed state. This latter state is thought to occur through a variety of intrinsic and extrinsic factors that consequently lead to a lack of adaptive immune cell responsiveness and T-cell and B-cell loss, due in part to apoptosis [12]. Unfortunately, new therapeutics to treat sepsis and biomarkers that may be used to identify those patients who are at the highest risk for developing later infections, and thus might best benefit from such therapies, are limited or lacking. BTLA is a co-inhibitory, immune-regulatory receptor, whose primary role in CD4^+^ T cells is thought to be maintaining a state of immune tolerance to self-antigens [35] or resolving (suppressing) proinflammatory responses [31]. However, such tolerogenic activity may inadvertently provide an immune-suppressed environment that permits pathogens, such as plasmodium [36], cytomegalovirus [37], and the intracellular bacterium *Listeria monogenes*[38], to thrive (at least under experimental conditions), while also unintentionally reducing tumor (cancer) cell immune surveillance by supporting a sustained state of immune suppression [39,40].

Recently, we reported that BTLA expression on myeloid cells contributes to experimental septic mortality in mice [28]. Here, we expand on this finding by demonstrating that BTLA was not only upregulated on mouse lymphocytes and contributed to cellular apoptosis in primary and secondary lymphoid organs that associated with CD4^+^ T-cell and B-cell loss, but also that BTLA was seen at a higher frequency on CD4^+^ lymphocytes in the SIRS patients that developed subsequent infections. These findings therefore potentially add immune dysfunction as mediated through lymphoid cell loss during SIRS and/or sepsis to the above list of disease states to which augmented BTLA expression appears to contribute.

Although there appear to be promising biomarkers for identifying which critically ill patients are septic (examples being procalcitonin, C-reactive protein and soluble triggering receptor expressed on myeloid cells, amongst others [6,7,41,42]), currently the biomarkers that delineate which critically ill patients are most susceptible to becoming septic are lacking. Here we find that an increase in the percentage of BTLA^+^CD4^+^ T cells is much higher in the patients that develop subsequent nosocomial infections, thus implicating BTLA as a possible biomarker for identifying which critically ill patients are most susceptible to the development of sepsis. Only diminished levels of monocyte human leukocyte antigen-DR (mHLA-DR) have until now been reported as being an effective marker for identifying which critically ill trauma patients are susceptible to becoming septic [5]. An approach that measures both mHLA-DR and BTLA in assessing the critically ill patient’s susceptibility to sepsis may therefore be important for a number of reasons. First, the nature of the regulation of these receptors’ expression and their roles in contributing to immune suppression are very different. A decreased level of mHLA-DR on antigen-presenting cells is thought to represent a reduced capacity by these cells to activate the adaptive immune cells; however, increased BTLA expression on CD4^+^ T cells may represent a reduced ability for these cells to specifically function due to BTLA’s co-inhibitory molecule effects. Additionally, the concept of combining biomarkers has shown some potential, because a recent article demonstrating the potential value of combining multiple biomarkers (including procalcitonin, soluble triggering receptor expressed on myeloid cells and CD64 expression on neutrophils) for identifying and diagnosing sepsis in critically ill patients was found to be much more accurate than any one of these biomarkers alone [43]. A panel of biomarkers that includes the measurement of mHLA-DR on monocytes and BTLA on CD4^+^ T cells may therefore be similarly useful in accurately predicting a patient’s infectious susceptibility, if not their state of immune responsiveness.

In addition to our recent work demonstrating the importance of BTLA’s contribution to septic mortality and innate immune cell dysfunction during sepsis [28], the expression levels of BTLA and its ligand, herpes virus entry mediator, have also been evaluated in patients who had died from sepsis in a recent study by Boomer and colleagues [13]. Interestingly, this group found that the percentage of BTLA^+^CD4^+^ cells in patients who succumbed to sepsis were not significantly different from those who died from causes other than sepsis (both were at ~60 to 70% BTLA^+^); however, patients who died from lung cancer had lung CD4^+^ T cells that were increased to >80% BTLA^+^[13]. The contrast in these results from postmortem sampling to the live patient data we present here suggests that there may not only be differences in the level of circulating blood CD4^+^BTLA^+^ versus their lung CD4^+^ T-cell sample sites, but also in the timing of the sample acquisition during the sepsis process. Another possibility is that, given very few of our patient population died, BTLA expression levels may change as patients are closer to death. Considering we found that BTLA^-/-^ mice were protected from lymphoid cell apoptosis, it is also possible that many of the BTLA^+^CD4^+^ T cells in Boomer and colleagues’ study had already undergone apoptosis and were lost by the time these patients had succumbed to sepsis (compared with our data, in which we see a high level of circulating BTLA^+^CD4^+^ lymphocytes in septic patients).

Amongst BTLA, induction of other co-inhibitory receptors, including PD-1 and CTLA-4, have been demonstrated to contribute to CD4^+^ T-cell apoptosis following CLP in mice, as well as in septic patients [13,24,25]. In contrast to the mouse studies evaluating PD-1 and CTLA-4 in sepsis, we found that BTLA in mice was constitutively expressed on a very high percentage of CD4^+^ T cells, but the intensity of this expression was further increased in response to CLP, while CD4^+^ T cells only increased in the percentage of CTLA-4^+^ and PD-1^+^ expressing lymphocytes in response to CLP [24,25]. In critically ill patients with septic shock, an increase in PD-1^+^CD4^+^ lymphocytes when compared with healthy volunteers has also been observed [26,27]. Interestingly, a higher percentage of PD-1^+^CD4^+^ T cells correlated with a lower proliferation rate of CD4^+^ T cells [27] and blockade of the PDL-1:PD-1 pathway *ex vivo*, preventing CD4^+^ T-cell apoptosis [26]. However, neither study looked at whether PD-1 or CTLA-4 was induced in nonseptic, SIRS critically ill patients. Thus, it remains to be established whether PD-1 and/or CTLA-4 are also induced by critical injury alone prior to septic infection, like BTLA, or whether they act in a more redundant manner during sepsis to cause further dysfunction and/or apoptotic loss of these cells.

Interestingly, these data also suggest that BTLA in human CD4^+^ T cells has the capacity to induce the mobilization of these cells into circulation, which appears to contrast the findings that we and others have observed in mice. Alternatively, however, it has been demonstrated that the cross-linking of BTLA on human CD4^+^ T cells leads to an inability to be fully activated (via upregulation of CD25), while also inhibiting their capacity to secrete interleukin-2, interleukin-4, interleukin-10 and interferon-gamma [44], which appears to be consistent with BTLA in mice [18]. Therefore, BTLA expression on CD4^+^ T cells may help to drive the cells into circulation in an exhausted state with a diminished capacity to be activated; however, further studies are needed to provide evidence for this hypothesis.

This study is of course not without limitations. Overall, prior to understanding whether using a BTLA antagonist is a viable option for the treatment of critically ill patients, additional studies are needed to elucidate whether the augmented BTLA expression that we observed on the septic and SIRS patient and mouse CD4^+^ T cells indeed inhibits their function (including *ex vivo* cytokine production and upregulation of activation markers, such as CD69 and CD25), proliferation, or differentiation. Also, with regard to BTLA as a biomarker for subsequent infections, significantly larger patient numbers need to be collected to establish the specificity and sensitivity with which BTLA expression can be used to predict the severity of disease (that is, sepsis, septic shock or severe sepsis), septic mortality, multiple organ damage, and to what particular pathogens these patients may be susceptible. Additionally, a previous study by Zhang and colleagues found that the percentage of BTLA^+^CD4^+^ T cells from healthy volunteers was much higher (~90% double positive); thus, future studies must be conducted with similar methods to those of Zhang and colleagues (that is, staining technique and a similar BTLA antibody clone) to determine whether SIRS patients that develop a subsequent infection have diminished BTLA expression with alternative staining methods such as these[32]. This would further clarify the use of BTLA as a potential biomarker. Another limitation of this study was that, because of the relatively small sample size, patient samples were compared at various times following ICU admission. Although we properly compared these ICU patients based on whether they were SIRS or septic, sampling at specific times following ICU admission and septic insult would provide more specific information about the timing of changes in BTLA expression on CD4^+^ T cells during SIRS and sepsis. Additionally, in our mouse data that evaluated apoptosis against CD4^+^ T-cell and B-cell loss, only an indirect association of the contribution of BTLA expression to the apoptotic cell loss of CD4^+^ T cells and B cells could be drawn. Future studies, such as ones that directly evaluate whether CD4^+^ T cells and B cells are protected from the induction of apoptosis (with markers such as activated caspase-3 and Annexin-V) in BTLA^-/-^ mice compared with WT mice, should be carried out to confirm our results. Finally, studies that adoptively transfer BTLA^-/-^ CD4^+^ T cells into WT mice may be able to elucidate whether BTLA expression on CD4^+^ T cells inappropriately drives an exhausted form of CD4^+^ T cells into circulation during experimental sepsis.

## Conclusions

We feel that the data presented here concerning BTLA expression on CD4^+^ T cells in critically ill ICU patients and experimental mice importantly add novel insight as to how ligation of BTLA may contribute to the induction of the adaptive immune cell loss that is associated with sepsis. Further, these data illustrate the value of BTLA as a potential therapeutic target and a possible biomarker for identifying the critically ill patients whom are most susceptible to develop subsequent or secondary infections, and thereby may allow for the delivery of the sufficient therapies that are needed to help prevent the morbidity and mortality associated with SIRS and sepsis.

## Key messages

• The percentage of BTLA expressing circulating peripheral blood CD4^+^ T cells was higher in septic critically ill patients when compared with SIRS ICU patients and healthy controls.

• An increased percentage of BTLA^+^CD4^+^ T cells in critically ill patients associated with poor outcomes and a longer hospital length of stay; thus, future studies are warranted examining BTLA expression on CD4^+^ T cells as a potential biomarker for subsequent infections and poor septic outcomes.

• BTLA expression was induced on CD4^+^ T cells and B cells following experimental septic injury in mice.

• Lack of BTLA protected primary and secondary lymphoid organs from cellular apoptosis, and this was associated with the loss of CD4^+^ T cells and B cells following CLP in mice, suggesting that BTLA may be directly involved in CD4^+^ T-cell apoptosis during experimental sepsis.

## Abbreviations

BTLA: B and T lymphocyte attenuator; CD4: Cluster of differentiation 4; CLP: Cecal ligation and puncture; CTLA-4: Cytotoxic T-lymphocyte antigen-4; MFI: Mean fluorescence intensity; mHLA-DR: Monocyte human leukocyte antigen-DR; PD-1: Programmed death receptor-1; SIRS: Systemic inflammatory response syndrome; TUNEL: Terminal deoxynucleotidyl transferase; WT: Wild type.

## Competing interests

The authors declare that they have no competing interests.

## Authors’ contributions

NJS, C-SC and AA conducted the general study concepts and design and wrote the manuscript. All of the experiments were performed by NJS. DSH and SFM substantially contributed to the design and conception of the human studies. DSH obtained the Institutional Review Board approval for the human subjects study. All authors have given final approval for this manuscript to be published.
